# Treatment of longitudinal stent compression under intravenous ultrasound guidance: A case report

**DOI:** 10.1097/MD.0000000000009405

**Published:** 2017-12-22

**Authors:** Daoyuan Si, Hongliang Yang, Guohui Liu, Yaliang Tong, Yuquan He

**Affiliations:** Department of Cardiology, China-Japan Union Hospital of Jilin University, Changchun, China.

**Keywords:** Complication, longitudinal stent compression, percutaneous coronary intervention

## Abstract

**Rationale:**

Longitudinal stent compression is a rare phenomenon, which has been increasingly reported in recent years.

**Patient concerns:**

Following 2 stents implanted into the middle and proximal segments of the left anterior descending (LAD) artery, longitudinal stent compression occurred when a post-dilation balloon was introduced into the proximal stent opening.

**Diagnose:**

The intravenous ultrasound (IVUS) examination revealed overlapping at the opening of the proximal stent and poor stent adherence.

**Interventions:**

Another balloon was carefully inserted into the opening for post-dilation, followed by angiography and IVUS examination.

**Outcomes:**

The IVUS examination indicated that the overlapping at the opening of the proximal stent was improved and the stents were well adhered.

**Lessons:**

Such compression may be prevented by gentle and careful balloon maneuverability and improved with the use of additional balloon angioplasty or stent implantation.

## 1 Introduction

Longitudinal stent compression is a rare complication after coronary stent implantation. However, it has been increasingly reported in recent years, with the design of new thin stents, and the development of radiographic technology and stent radio-opacity. In fact, its occurrence may be associated with equipment operation. Here, we present a case of equipment-related longitudinal stent compression to raise awareness of this complication. We also present effective methods to prevent and manage longitudinal stent compression.

## 2 Case presentation

**Figure 1:**
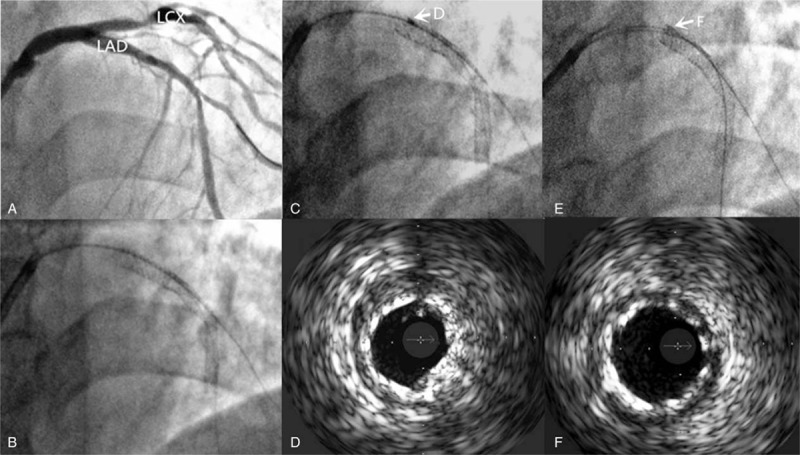
Percutaneous intervention of left anterior descending (LAD) artery. (A) 70%, type A lesion of medial LAD. (B) Two stents placement. (C) Longitudinal compression of the proximal stent as marked by a white arrow. (D) Intravenous ultrasound was performed immediately after longitudinal compression. (E) Post stent deployment, the longitudinally compressed proximal part of stent as marked by a white arrow. (F) Intravenous ultrasound was performed after dilation.

A 60-year-old female patient with a 7-year history of chest pain but no history of hypertension or diabetes was hospitalized after experiencing exacerbation of pain for 2 months. Electrocardiography showed no significant ischemic changes. Selective coronary angiography revealed a long stenosis in the proximal to medial segment of the left anterior descending (LAD) artery. The narrowest portion and medial segment of the left circumflex (LCX) artery were approximately 70% stenosed (Fig. 1A). The fractional flow reserve values calculated from the pressure measurement were 0.87 and 0.76 for the LCX and LAD, respectively. A pressure gradient (13 mmHg) was detected in the medial portion of LAD after the guidewire was withdrawn.

For stent placement, a BMW guidewire (Guidant Corp, Indianapolis, IN) was first inserted into the distal LAD and the lesion in the proximal to middle segments was dilated using a 2.0 × 20 mm Voyager balloon (Abbott Vascular, Santa Clara, CA). 2.5 × 28 mm and 3.0 × 16 mm Element stents (Boston Scientific Co., Natick, MA) were placed at the medial and proximal lesions, respectively, followed by post-dilation with a 3.5 × 12 mm Voyager NC balloon (Abbott Vascular, Santa Clara, CA) (Fig. 1 B). However, slight compression resistance occurred when the balloon was first introduced into the LAD because the head end of the balloon reached the opening of the proximal stent (Fig. 1C). At this point, the balloon had to be withdrawn and the guidewire position was slightly adjusted so that another balloon could be carefully maneuvered past the proximal stent without difficulty. The overlapping stents were then post-dilated and examined by radiography.

The results demonstrated stent malapposition in the proximal anterior descending artery and longitudinal stent compression at the opening with shortening on one side and curled upward on the other side. An intravenous ultrasound (IVUS) catheter was also inserted into the stent. The IVUS examination also revealed overlapping at the opening of the proximal stent and poor stent adherence (Fig. 1D). After dilation using a 3.5 × 8 mm GRIP balloon (Acrostak, Winterthur, Switzerland) inflated to 20 atm, angiography showed that the longitudinal stent compression at the opening of the proximal stent was markedly improved as evidenced by a thrombolysis in myocardial infarction grade 3 flow (Fig. 1E). A second IVUS examination indicated that the overlapping at the opening of the proximal stent was improved and the stents were well adhered (Fig. 1F). The patient did not show any adverse symptoms such as chest pain during the surgery. At 5 days after surgery, the patient was discharged. The patient complained of no discomfort during the 1-year follow-up period. The study was approved by the Ethics Committee of China-Japan Union Hospital of Jilin University, China.

## 3 Discussion

Longitudinal stent compression refers to the reduced length of the deployed stent upon longitudinal compression and is a rare complication after coronary stenting. Its occurrence may be related to tortuous vessel-related lesions, calcified lesions, chronic total occlusion, open lesions, and lentigo maligna lesions. In addition, improper operating practices such as instruments including post-dilation balloon and IVUS catheters passing through a deployed stent, squeezing a stent by deeply inserting a catheter, squeezing a guiding catheter over a stent upon balloon withdrawal, and distal stent placement through a proximal stent (particularly when the proximal stent poorly adheres) may be responsible for the longitudinal stent compression.^[1,2]^

Contemporary stents generally have thinner cylindrical bodies that enable better passing and compliance, but this feature inevitably decreases its longitudinal strength and stability. Longitudinal stent compression has been increasingly reported, with the improvement of radiographic technology and the increase of stent radio-opacity. The Promus stent has a relatively high occurrence of longitudinal stent compression, though isolated report of other stents such as Taxus Liberte (Boston Scientific Co., Natick, MA), Biomatrix (Biosensors Interventional Technologies, Singapore), Endeavor (Medtronic Inc., Minneapolis, MN), and Xience (Abbott Vascular, Santa Clara, CA).^[1,3]^ The Promus stent was also used in the case reported here. Compression that occurred in its proximal portion because of slight compression during balloon post-dilation was confirmed by coronary angiography and IVUS examination. However, during the postdilation process, contact between the instruments and the stent was inevitable because the proximal anterior descending artery had a certain arc and the guidewire had to remain close to the proximal stent.

Ormiston et al^[4]^ and Prabhu et al^[5]^ compared the longitudinal compression performance of 4 different commercial stent design families and found that the Promus stent, with its 2-link offset peak-to-peak design, has the lowest compression resistance compared to the other stent design families and that its shortening is closely associated with its low longitudinal strength. Its relatively high radio-opacity can make it easy to be detected.^[6]^ Although there is no evidence showing that longitudinal stent compression can adversely affect patient prognosis, care must be taken during surgery to avoid stent shortening. One should ensure the following: proper management of the target lesion (pre-dilation) before stent placement; good stent adherence for stent placement; insertion of the guiding catheter to the appropriate depth; minimizing of guidewire deviation away from the lumen center; sufficient inflation and withdrawal time of post-dilation balloon catheters, especially those with large diameter; implantation of the distal stent first for overlapping stent placement; minimizing of guidewire deviation and the movement of assisted instruments through the stent when resistance occurs in the process only to the point of resistance; and the use of additional balloon angioplasty or stent implantation to improve stent adherence and cover lesions area as much as possible if longitudinal stent compression occurs.

In the case reported here, IVUS revealed that the adherence of proximal stent was poor, which may make the postdilation balloon easy to reach the stent. However, when longitudinal stent compression occurred, timely adjustment of the guidewire position and the gentle and careful introduction of another balloon into the proximal stent for post-dilation markedly improved the poor stent adherence and shortening.

## 4 Conclusion

Longitudinal stent compression is a rare phenomenon that may occur during the implantation of thin cylindrical stents, especially Promus stents, probably because of their design and high radio-opacity. Such compression may be prevented by gentle and careful balloon maneuverability and improved with the use of additional balloon angioplasty or stent implantation.
